# ‘It won’t catch us off guard this time’: interview study exploring use of mental health care plans for birth and the postpartum period within perinatal mental health services

**DOI:** 10.1192/bjo.2026.12019

**Published:** 2026-06-24

**Authors:** Cornelia Carey, Rona Hunt, Catherine Hinds, Chai Jairaj, Nuala B. Kane

**Affiliations:** Perinatal Mental Health Service, The National Maternity Hospital, Dublin, Ireland; Perinatal Mental Health Service, The Coombe Hospital, Dublin, Ireland; Department of Psychiatry, Trinity College Dublin, Dublin, Ireland; Department of Psychiatry, University College Dublin, Dublin, Ireland

**Keywords:** Perinatal mental health, advance care planning, lived experience, having a voice, individualised care

## Abstract

**Background:**

Evidence shows that advance care planning has the potential to reduce involuntary admissions and empower service users. The perinatal period is a time of heightened risk of relapse of mental illness, and, in this context, many perinatal mental health services routinely offer pre-birth mental health care planning meetings.

**Aims:**

We aimed to explore the experience of perinatal mental health service users and their partners following a pre-birth planning meeting and the writing of a perinatal care plan that included advance care plans for birth, postpartum and in case of crisis.

**Method:**

We interviewed pregnant perinatal mental health service users and their partners at two large, urban maternity hospitals in Dublin, Ireland. We used thematic analysis to identify key themes relevant to their experiences of pre-birth planning meetings and written perinatal mental health care plans.

**Results:**

Ten service users and three partners were interviewed. We identified five themes: theme 1, Hoping for change; theme 2, A wish to be heard; theme 3, Individualised care; theme 4, Security of ‘a plan in place’ and theme 5, Role of the support network.

**Conclusions:**

Women and their partners value pre-birth planning meetings and these should routinely be offered within services, with consideration as to the size and timing of the meeting, and who is in attendance. These findings are relevant to general adult and liaison psychiatrists who should also incorporate advance care planning into routine practice.

In perinatal mental health services, advance care planning, through development of a perinatal mental health care plan at a pre-birth planning meeting (PBPM), is a widely accepted clinical practice endorsed by clinical guidelines.^1^ The perinatal mental health care plan is a non-binding document developed collaboratively by the perinatal mental health team and the pregnant service user. It outlines the woman’s wishes and their team’s recommendations for their mental healthcare before, during and after the birth of their baby, and what to do in a mental health crisis. The ‘Pan-London Perinatal Mental Health Networks Pre-birth planning: Best Practice Toolkit for Perinatal Mental Health Services’^2^ sets out guidance for conducting PBPMs. This includes who to invite, what to discuss at the meeting and which areas to include in the perinatal mental health care plan (including antenatal care, the maternity admission, the postnatal period and crisis planning). However, despite guidance being in place,^2^ there is limited supporting literature on PBPMs to date,^3,4^ and no current empirical data on views and experiences of stakeholders.^5^

Evidence outside the perinatal context shows that mental health advance care plans can empower mental health service users,^6,7^ improve therapeutic relationships^8,9^ and reduce involuntary admissions.^10,11^ A recent Irish survey of in-patient psychiatry service users found that most would be interested in making an advance directive and would involve a psychiatrist, and that half preferred non-binding directives.^12^ However, unlike in perinatal services, and despite this evidence, uptake of advance care planning in wider mental health services remains poor.^13^

Advance care planning may be particularly pertinent to perinatal mental health service users for several reasons. First, birth is a predictable future event and a known risk factor for relapse of serious mental illness.^14^ The risk of relapse in women with bipolar disorders has been estimated at 8–25% in some studies;^15,16^ reports from tertiary perinatal mental health clinics are much higher at 71%, and higher again during pregnancy for those who discontinue medication (86%).^17^ An episode of postpartum psychosis is a psychiatric emergency, can quickly become severe and the presentation can vary substantially from hour to hour.^18^ There is also a significant relapse risk for childbirth-related post-traumatic stress disorder,^19^ and collaborative informed decision-making has been recommended following traumatic experience of childbirth.^20^ Second, a study by Bipolar UK showed that women are acutely aware of the risks in pregnancy and the postpartum period, and so it might present a window of opportunity to engage women in their mental healthcare.^21^ Finally, pregnant women face many decisions, including whether to stop or change their medication during pregnancy and postpartum,^22^ and how best to plan for birth and the postnatal period.^15,23,24^

With this in mind, our study aimed to explore the views and experiences of perinatal mental health service users, and their supporters, who had attended a PBPM and developed a perinatal care plan, on this process. It is our view that examining the practice of perinatal mental health care planning has the potential to generate insights relevant to advance care planning both in perinatal services and across wider mental health services.

## Method

### Study design

This was a single point-in-time interview study of perinatal mental health service users (and their supporters) who attended PBPMs at two large, urban maternity hospitals (The National Maternity Hospital (NMH) and The Coombe Hospital in Dublin, Ireland). Thirteen semi-structured in-depth interviews of ten service users and three supporters were conducted between November 2024 and August 2025. Semi-structured interviews were chosen to permit natural deviation from the set interview schedule, to ask follow-up questions to points made by the participants, thus enriching the discussion and allowing for more in-depth exploration of those points.^25^

Reflexivity was actively considered throughout the research process. The researchers are all practicing psychiatrists with clinical experience of advance care planning; C.C. and R.H. were senior registrars in perinatal mental health services during the study (NMH and The Coombe Hospital, respectively), C.H. and C.J. are consultant perinatal psychiatrists (NMH and The Coombe Hospital, respectively) and N.B.K. is a consultant general adult psychiatrist and clinical researcher in mental health ethics and law. C.C., R.H., C.H. and C.J. conducted PBPMs at both hospitals during the study period, and this positionality was considered at data collection and analysis stages. To minimise the risk that participants might withhold negative views, the interviewers (C.C. and R.H.) did not interview participants whose care they were involved in. Triangulation and regular reflection were employed at the analysis stage, as described below.

The authors assert that all procedures contributing to this work comply with the ethical standards of the relevant national and institutional committees on human experimentation and with the Helsinki Declaration of 1975, as revised in 2013. All procedures involving human patients were approved by the NMH (approval number EC32.2024) and The Coombe Hospital (approval number 22-2024). The Standards for Reporting Qualitative Research (SRQR) guidelines^26^ were followed in reporting.

### Setting and PBPMs

The NMH and The Coombe Hospital are two of three maternity hospitals which serve the population of Dublin, Ireland. Both had approximately 6500 births in 2023.^27^ Both sites have separate perinatal mental health services that are consultant-led and delivered by a multidisciplinary team. Both services hold PBPMs guided by the ‘Pan-London Perinatal Mental Health Networks Pre-birth planning: Best Practice Toolkit for Perinatal Mental Health Services’.^2^ In both hospitals, PBPMs are usually held at 30 weeks gestation. They are routinely offered to service users with a history of severe mental illness (particularly bipolar disorder or psychosis) or eating disorders, for whom the PBPMs are often held earlier because of increased obstetric risks. PBPMs are sometimes offered to service users with other conditions, e.g. obsessive–compulsive disorder or tokophobia (phobia of childbirth), according to clinical judgement (for example, in cases of severe illness, complex medication regimes or significant risk concerns). For cases where service users are too unwell to attend, a professionals’ meeting is held instead, and a perinatal mental health care plan is developed in absentia. During the PBPM, a perinatal mental health care plan (see Supplementary Material 1) is completed with the service user, with the option to make amendments. It is given to the service user, added to their patient chart and shared with other supporters and clinicians as per the service user’s wishes.

### Recruitment and data collection

Eligible pregnant service users who attended PBPMs in the NMH and The Coombe Hospital perinatal services over the study period were offered the opportunity to participate in the study. Service users were excluded from recruitment if they were (a) under 18 years, (b) non-English speaking, (c) had an intellectual disability, (d) were a current psychiatric in-patient or (e) if the meetings involved serious child protection concerns necessitating involvement of Child and Family Agency social workers (as these were mandatory meetings focused on child protection concerns). We also excluded service users who lacked capacity to consent to the study as assessed by their treating clinician.

Following the PBPM, service users were informed of the study by their treating clinician, who also provided the patient information leaflet. Service users were asked if an investigator could contact them by phone or email to discuss the study further. They were advised that participation in the study made no difference to their clinical care and that they could withdraw at any time. If they wished to participate, service users signed a written consent form. If a service user consented to participate in the study, they were asked if a supporter was present and whether they could be invited to partake. If the service user consented to this, the supporter was contacted and offered the opportunity to participate. Recruitment was ceased when data saturation was reached.

Interviews were conducted by R.H. (*n* = 5) and C.C. (*n* = 8) by video call within 2 weeks of the PBPM. They explored the views and experiences of interviewees in relation to the PBPM and creation of the mental health care plan. The interview protocol (see Supplementary Material 2) was adapted to the current perinatal context from a previous study.^28^ Demographic information, details of previous psychiatric and pregnancy history and current diagnosis were collected from service user charts to supplement and contextualise the qualitative interview data.

### Data analysis

C.C. and R.H. recorded and transcribed interviews electronically, cleaned transcripts and uploaded these to the qualitative data analysis software package NVivo Version 12 for Windows (Lumivero, Denver, Colorado, USA; https://lumivero.com/products/nvivo/). C.C. and R.H. anonymised and collated data for analysis. R.H., C.C. and N.B.K. conducted thematic analysis, in keeping with Braun and Clarke’s methodology.^29^ We chose Braun and Clarke’s reflexive thematic analysis because of its systematic and rigorous approach and its adaptability across disciplines, allowing description and interpretation of rich qualitative data. C.C. and R.H. coded 13 transcripts, and N.K. coded 5 transcripts for triangulation. First, C.C., R.H. and N.B.K. identified relevant words and phrases in all transcripts, generating and applying codes. They then met to discuss and combine these codes into an initial thematic map including semantic (explicit or surface level meaning) and latent (underlying meaning) themes. They then reviewed this thematic map for coherence within and between themes, engaging in repeated discussion and refinement of themes until the final set of themes was produced.^30^ Given the positionality of the researchers, all but one of whom conducted PBPMs, discussions incorporated reflection on how the researcher’s own experience contributed to their interpretation as well as the risks of privileging positive over negative views of the process.

## Results

### Sample characteristics

Twenty-three PBPMs that fit the selection criteria were held across the two hospitals within the time period. Of those, ten service users were recruited, six attending the NMH and four attending The Coombe Hospital. Of those not recruited, eight service users declined to partake in our study, three were uncontactable and two delivered pre-term. Thirteen interviews were conducted, including the ten service users and three supporters (of six supporters who attended eligible PBPMs). All supporters were male partners of the interviewees.

Table 1 outlines characteristics of included service users. Participants had a median age of 33 years (range 28–41 years). Median gestational age at time of care planning meeting was 32.5 weeks (range 20–36 weeks). All service users reported no previous experience of advance care planning.

**Table 1:** Characteristics of the ten women interviewed Table 1 long description.

Characteristics	Frequency (*n* = 10)
Age, years	
20–29 (%)	1 (10)
30–39 (%)	7 (70)
≥40 (%)	2 (20)
Ethnicity	
White, Irish (%)	7 (70)
White, European (%)	2 (20)
Black (%)	0 (0)
Asian (%)	1 (10)
Marital status	
Married (%)	8 (80)
Cohabiting (%)	2 (20)
Single (%)	0 (0)
Pregnancy status	
Nulliparous (%)	5 (50)
Parous (%)	5 (50)
Diagnosis	Previous postpartum psychosis (*n* = 1)
	Schizophrenia (*n* = 1)
	Bipolar affective disorder (*n* = 2)
	Anorexia nervosa (*n* = 2)
	Perinatal obsessive–compulsive disorder (*n* = 2)
	Previous birth trauma with secondary tokophobia (*n* = 2)
PBPM format	In person (*n* = 7)
	Online (*n* = 3)
PBPM attendance	Partners attended (*n* = 6)
	Staff outside perinatal mental health team attended (*n* = 3)

PBPM, pre-birth planning meeting.

### Themes

The thematic analysis of women and their partners’ experiences of PBPMs generated five key themes: theme 1, Hoping for change; theme 2, A wish to be heard; theme 3, Individualised care; theme 4, Security of ‘a plan in place’ and theme 5, Role of the support network.

Titles of themes and subthemes are presented in Table 2. A thematic map with further illustrative quotes is included in Supplementary Material 3.

**Table 2:** Themes and subthemes Table 2 long description.

Themes	Subthemes
1. Hoping for change	Experience motivating planning
	Parenthood motivating planning
	Avoidance
2. A wish to be heard	Valuing feeling listened to in the meeting
	Written care plan carrying the woman’s voice
	Plan in the obstetric context
3. Individualised care	Individualised care valued by women
	Negatives of individualised care – burden and stigma
4. Security of ‘a plan in place’	Making the plan
	Specifics of the plan
	Timing of the meeting
5. Role of the support network	Personal supports
	Professional supports (and communication)

#### Theme 1: Hoping for change

This included three subthemes: Experience motivating planning, Parenthood motivating planning and Avoidance.

##### Experience motivating planning

‘You’re thinking back on your previous experience, “OK well, this happened and maybe this time I’d like this instead”. It’s causing you to think again, whereas you might not have done it in a long time, and it is a bit of a relief.’ (SU 10, secondary tokophobia)

Participants and their partners described how difficult their past experience of mental illness had been, particularly if it occurred in the postpartum period. For some participants and their partners this served as a motivator to remain well and to actively engage in care planning, e.g. ‘It won’t catch us off guard this time’ (Partner 4, postpartum psychosis). Participants and their partners often expressed hope for a different experience going forward.

##### Parenthood motivating planning

‘I have children [so] I would be reluctant for them to be around me if I was very unwell. I wouldn’t like them to be exposed to [that], as much as I love to see my kids and I found it very hard when my son was a baby and I was basically gone for seven weeks overnight and that was horrific. [But] I would feel more guilt afterwards when I would be thinking “God, you saw me unwell” and that would be harder to carry afterwards.’ (SU 2, bipolar disorder)

Parenthood was a core focus for a number of participants, with service users describing real or imagined separation from their children, the guilt associated with their children witnessing an episode of illness and questioning their ability to mother while unwell. Many participants explicitly described their role caring for their children as a primary motivator to remain well. One supporter referred to the strain of balancing care for their unwell partner with care for the baby.

##### Avoidance

‘I was saying, “I’m just trying not to think about it”. Then [the doctor] was saying, “Sometimes that isn’t a useful thing to do”. So, I’ve been trying.’ (SU 5, perinatal obsessive–compulsive disorder)

‘And then you know you have to go back and discuss depression, medication, hypomania, which is crazy, right? So, you don’t even want to talk about these things because you’re not yourself. The care plan was very carefully discussed. I was told “Let’s put it on paper and it’s there. It doesn’t mean that it’s going to happen.”’ (SU 9, bipolar affective disorder)

In contrast to those whose past experience motivated them to plan, some participants described their past experience as leading to a reluctance to think about their illness. This acted as a barrier to thinking and talking about relapse risk. Some participants noted previous experience of illness and associated mental healthcare meetings as ‘daunting’. Some had pre-conceived ideas of what the PBPM would be like. At times, the meeting subverted the participants’ expectations; some highlighted an unexpected maternal focus and finding the meeting materially useful. A positive experience of care planning helped to alleviate the need for avoidance.

#### Theme 2: A wish to be heard

This included three subthemes: Valuing feeling listened to in the meeting, Written care plan carrying the woman’s voice and Care plan in the obstetric context.

##### Valuing feeling listened to in the meeting

‘We both have our opinions. I think that they were both well received and listened to, and there was real caregiving.’ (Partner 7, perinatal perinatal obsessive–compulsive disorder)

Most participants felt that they had been listened to in the PBPM, particularly if the meeting was small with just their treating clinician and a supporter. Partners also felt listened to within the meetings. Participants generally believed that the care planning meeting was designed for their benefit and to describe their experience of ill health rather than the psychiatrist’s perception of that experience.

##### Written care plan carrying the woman’s voice

SU 2 (bipolar disorder): ‘It’s nearly like, I know that it sounds strange, it’s nearly like a will, what your wishes are, after the event [previous postpartum relapse]’

Interviewer: ‘So it’s you being able to say what you would like, if you were in a position to?’

SU 2: ‘Exactly and that’s very important because when I am unwell, or anyone in that position […] we can’t talk through or you can’t vocalise in a normal sense what you would feel or want.’

Participants endorsed the written care plan as a facilitator for them having a voice at times when they would not be able to speak up for themselves, both in labour (e.g. ‘Just the fact that you don’t have to advocate, you know, in that moment for yourself’ (SU 10, secondary tokophobia)) and during an acute episode of their mental illness. One participant commented on the care plan containing exactly what had been discussed in the meeting. Another clarified that she would not want her partner to make decisions for her, but that she would trust him to use the care plan to advocate for her if she were incapacitated.

##### Care plan in the obstetric context

‘I was in with my consultant [obstetrician] today and she said “We’ll make sure that this is done as well”. I could even actually see in my notes today, like the care plan stuck in the front of it where it was said that it was going to be stuck.’ (SU 6, secondary tokophobia)

Participants valued the translation of voice to action with evidence of transparency and follow-through within the maternity hospital. One participant mentioned that her obstetrician had referred to the care plan in a subsequent clinic appointment. Most participants wished for their written care plan to be visible in their chart. Those in the maternity hospital using paper-based records commented on the importance of seeing their care plan in the chart. The practicality of a succinct written care plan was noted as instrumental in allowing busy maternity staff to attain an understanding of the woman’s situation quickly.

However, one participant noted that a succinct care plan did not adequately capture the complexity of their experience: ‘It kind of feels like it’s there’s a lot more to this. I can’t quite put my finger on it’s just not sitting right with me, just the whole… document. I don’t think it has covered everything’ (SU 3, anorexia nervosa). Another expressed concern about ‘whether [her care plan, which had multiple points] will be taken seriously by busy staff on the day’ (SU 9, bipolar disorder).

#### Theme 3: Individualised care

This included two subthemes: Individualised care valued by women and Negatives of individualised care – burden and stigma.

##### Individualised care valued by women

‘I actually thought it was very good because it was very specific to me, because I was afraid it would be very general.’ (SU 7, perinatal obsessive–compulsive disorder)

‘No, because you make it your own. You know, you tailor it yourself.’ (SU 10, secondary tokophobia)

Participants and their supporters valued that their care plan was specific to their own wishes and care needs, rather than being a generic mental health care plan. This approach to individualised care was materially useful in generating helpful suggestions and gave participants a sense of ownership over their own care plan.

##### Negatives of individualised care – burden and stigma

‘They’ll think you’re odd you know if there’s that space in your chart that other people would see.’ (SU 5, perinatal obsessive–compulsive disorder)

‘Obviously, there are people that are a lot worse than me and maybe still have an eating disorder very active at the moment, and I didn’t think that [it] would necessarily be the midwives’ job to ensure that you’re eating and support you in that sense.’ (SU 8, anorexia nervosa)

This kind of individualised care was not always endorsed as positive. One participant expressed a sense of stigma regarding their mental illness and the care plan being in their chart. Both participants with eating disorders expressed concern regarding the burden on maternity staff in having to address their mental health needs within a busy service.

#### Theme 4: Security of ‘a plan in place’

This included three subthemes: Making the plan, Specifics of the plan and Timing of the meeting.

##### Making the plan

‘[It] kind of helps you relax, I think, just knowing that there’s a plan in place… After the meeting, like both myself and my husband came out with a weight off our shoulders.’ (SU 4, postpartum psychosis)

Participants generally appreciated the process of the care planning meeting and the reassurance and security of having a plan in place, regardless of the specific details. Most appreciated the clinician’s ability to offer guidance and containment around the uncertain risk of relapse. Knowing who to contact and what options were available was valued above any form of guarantee, or even diagnostic clarity, in one case. However, not all participants felt reassured by their crisis plan and one described craving more guidance: ‘…[I was] hoping for a plan for [the] middle ground of when things start to go rather than waiting until I need a full-blown medical admission’ (SU3, anorexia nervosa).

##### Specifics of the plan

‘So, I just know that people will all be looking out for things and we included [early warning] signs to watch out for.’ (SU 4, postpartum psychosis)

‘[Medication is] nearly like an armour, you know that’s a protective thing [to] stop me getting that unwell.’ (SU 2, bipolar disorder)

Participants valued the use of early warning signs in their care plan. They also referred to the importance of sleep and specific practical adaptations within the maternity hospital. The integration of non-pharmacological interventions surprised service users.

Medication was specifically referenced by the majority of participants and their partners, with one participant describing it as ‘like a security blanket’ (SU 4, postpartum psychosis). Participants considered medication to play a central role in relapse prevention and described its importance in their previous recovery. Participants were reassured that they would have a choice in medication. This was particularly important given past experience of side-effects, concern regarding potential risks to the foetus, wish to breastfeed and participants preferring lower doses of medication where possible.

##### Timing of the meeting

‘I feel like maybe starting that a little bit earlier just to have that reassurance.’ (SU 6, secondary tokophobia)

Participants valued the preventative ethos of the PBPM and a number of participants stated that they would have preferred an earlier meeting, closer to 20 weeks gestation. Those who had a PBPM at 20 weeks were happier with the timing. One participant and her partner strongly endorsed the pre-conception planning meeting as an even more useful form of advance care planning, as before attending this they thought they might be advised not to conceive. Another partner stated that they would have liked the option of a pre-conception planning meeting.

#### Theme 5: Role of the support network

This included two subthemes: Personal supports and Professional supports (and communication).

##### Personal supports

‘My husband knows where it is so he can always check it …He really supported me during that time, and he was actually very interested in having that plan so that he can refer to it.’ (SU 9, bipolar disorder)

‘It was just myself. So, my husband is a bit reluctant to be involved. I feel for him because it would have been very difficult for him when I ended up in the hospital the last time.’ (SU 2, bipolar disorder)

‘My partner actually wanted to come to it …and I was like there’s no need, you’ll be laughed at or told not to come in.’ (SU 8, anorexia nervosa)

Partners had varying roles in prevention and care. In two cases, the previous episode(s) of illness had created difficulties in communication with supporters, e.g. one participant described their partner as being unable to attend, given the trauma of the past experience of the service user’s mental illness, whereas another discouraged her partner from attending. The written care plan was a useful way for partners to provide support and to uphold the service user’s will and preference, even if they had not been able to attend the meeting.

##### Professional supports (and communication)

‘I thought it was better the midwife knows I’m having some problems. They told me they will help if any problems come up.’ (SU 1, schizophrenia)

A number of participants referred to the importance of having a therapeutic rapport with the clinician conducting the PBPM. Participants and their partners referred to a sense of safety and security particularly when that therapeutic relationship was pre-existing.

Most participants preferred a small meeting with the trusted clinician and for the written care plan to facilitate wider interdisciplinary communication. This included staff within the maternity hospital as well as external agencies and the participant’s general practitioner. However, some participants valued the care planning meeting itself as an opportunity for interdisciplinary communication ‘to have people in the same room and just make sure that everyone’s on the same page’ (SU 3, anorexia nervosa).

## Discussion

### Main findings

To the best of our knowledge, this is the first study to explore the experience of women and their partners undergoing pre-birth mental health care planning in perinatal services. Our study suggests that women value PBPMs and making an advance care plan for pregnancy, birth and the postpartum period. We found that women attending perinatal services, often with distressing past experiences of illness or childbirth, have a sense of hope that things can be different in future. This, along with concerns about parenting, provides a strong motivator to plan for future care during the perinatal period. The women and partners in our study expressed their wish to be active participants whose voices are heard by mental health and obstetric staff. Many valued the individualised care inherent in perinatal care planning, with a focus on their own relapse indicators and needs, whereas others were more ambivalent, expressing concern about the burden on staff and the possibility of stigma arising from an individualised care plan. Women and their partners highlighted that having ‘a plan in place’ can provide security and reassurance in itself, but also through planning for specific interventions such as medication and psychosocial supports. The importance (and sometimes, the complexity) of the woman’s personal and professional support network was evident, as well as how perinatal care plans could optimise communication across this network.

### Benefits perceived by service users and supporters

Our findings on perceived benefits of perinatal care planning chime with broader literature on service user views on advance care planning in wider mental health services. A recent systematic review identified that the main personal benefit for service users is an increased sense of empowerment, with advance directives described by service users as giving them ‘a voice’ in their care,^6,31^ similar to the women in our study. This review also highlighted repeated qualitative findings that advance directives can provide an increased sense of safety and ‘peace of mind’,^6,32^ echoing our finding of the security of having a plan in place. The review identified the potential for advance directives to enhance communication and continuity of care,^6^ a benefit also raised in our study. Although the wider literature also describes protection of service user rights and prevention of psychiatry-related harms as perceived benefits of advance directives,^6^ these aspects did not feature in our study, perhaps reflecting that perinatal care planning is a joint process instigated by the treating team, involving care plans that are not legally binding.

Our findings also suggest that PBPMs align with general recommendations for perinatal services, including optimisation of communication between obstetric and psychiatric services^33,34^ and provision of trauma-informed care.^35^ Our participants noted that the written care plan strengthened interdisciplinary working and was referenced by maternity staff, supporting previous claims that perinatal advance care planning could facilitate links between various stakeholders and promote more comprehensive care.^4^ The written care plan offered transparency and demonstrated follow-through of the care planning process across services. Personalised perinatal care that respects the individual needs of the patient is a standard set out by the Perinatal Quality Network^36^ and has been advocated for by The National Women’s Council of Ireland.^37^ This individualised approach to both pharmacological and psychosocial interventions inherent to perinatal care planning was noted and valued by our participants. Many participants spontaneously endorsed the role of medication in relapse prevention, showing consistency with previous findings that psychiatric advance directives are typically compatible with practice standards.^38^

### Concerns held by service users and supporters

Some participants described concern regarding stigma, particularly with the existence of a perinatal mental health care plan in their chart; similar concerns were raised in studies outside the perinatal context.^39^ Some participants questioned the burden that their care plan might place on a fast-paced maternity hospital, although they also valued knowing that maternity staff had access to their care plan. One service user expressed concern that her written care plan had not captured the complexity of her case, and that the crisis plan did not provide sufficient certainty or adequate options for early intervention. Another raised concern that in a very busy environment, staff might not have time to take the plan seriously; wider literature similarly reflects worries about advance directives not being followed.^6^ Although numbers were very small, and as such, between-group comparisons not possible, we noted that participants with anorexia nervosa appeared to express more ambivalence about the care planning process.

We note that the range of concerns expressed by these participants are less than that noted in the wider literature on advance decision-making.^6^ This may be because perinatal mental health care planning is initiated by the service rather than the service user, and so service users may have lower expectations of the process to begin with. Also, as noted later, those who declined to partake in the study may have had more negative views.

### Clinical and research implications

Our study has several clinical implications: first, perinatal services should routinely offer PBPMs as these are generally valued by service users and their supporters and offer several perceived benefits. The written care plan lends transparency to the process, facilitates interdisciplinary working, and is best kept short and practical. Perinatal services should take note that some women preferred meetings that were smaller and/or held earlier in pregnancy, and that some also endorsed the value of planning meetings during the preconception period. Second, wider mental health services should note how perinatal services have successfully incorporated advance care planning into routine clinical practice and consider following their example. Although there are various models of advance care planning, including psychiatric advance directives and crisis cards, we would consider PBPMs to be similar to the process of joint crisis planning common in jurisdictions such as Germany.^40^

Our study indicates the need for further research into several aspects of perinatal care planning. First, although the transdiagnostic nature of our sample was important in identifying common themes, there have been calls for research on perinatal psychiatric advance directives for specific diagnostic groups,^4^ and it would be useful to identify wishes and concerns specific to women with particular diagnoses. Second, exploring both psychiatric and maternity/obstetric staff perspectives of the process would be useful. Third, further exploration of the role of primary supporters is merited based on our findings. Fourth, preconception planning as a form of advance care planning has been endorsed both in clinical guidelines and by participants in this study and merits further investigation. Fifth, it would be very interesting to interview service users in the postpartum period, to explore their retrospective views of how the care plan functioned during and after birth. Finally, given that advance healthcare directives for mental healthcare are now legally binding in Ireland, it would be interesting to study how the perinatal care planning process could incorporate the writing of a binding advance directive.

### Limitations

This was a small study of ten women and three partners; however, we felt that the range of diagnoses in our sample accurately reflected the diagnostically heterogenous population under the care of perinatal services. Unfortunately, our study had no Black or transgender participants, no single parents and no same-sex couples; ensuring greater sociodemographic diversity should be a consideration in future studies. It was not possible to compare characteristics of participants and non-participants, as we were unable to collect data on the latter group. Participants who declined to partake in the study may have been more likely to be dissatisfied with the care planning process.

Four of the five authors (C.C., R.H., C.J., C.H.) were involved in delivery of psychiatric care in the two perinatal services studied. To minimise social desirability bias, we ensured that interviewers were not involved in the participants’ care. To minimise bias during data analysis, all transcripts were coded by at least 2 researchers (C.C. and R.H.), with 5 out of 13 transcripts also coded by N.B.K., who had no clinical role in perinatal services; these included any for which C.C. or R.H. had conducted the service user’s PBPM.

In conclusion, PBPMs offer an opportunity for perinatal mental health service users to express and record their will and preferences in relation to future mental healthcare during a transformative period of their lives. They also offer a formal opportunity to involve other stakeholders such as partners or other supporters. Our study shows that women value PBPMs and making an advance care plan; it carries their voice, gives them security, promotes individualised care and streamlines communication.

## Supporting information

10.1192/bjo.2026.12019.sm001 Carey et al. supplementary material Carey et al. supplementary material

## Data Availability

The data that support the findings of this study are available from the corresponding author, N.B.K., upon reasonable request.

## Supplementary material

The supplementary material is available online at https://doi.org/10.1192/bjo.2026.12019

## Table 1 Long description

The table presents characteristics of ten women interviewed, detailing their age distribution, ethnicity, marital status, pregnancy status, diagnosis, and PBPBM format. The age groups are 20-29 years, 30-39 years, and 40 years or older, with frequencies of 10 percent, 70 percent, and 20 percent respectively. Ethnicity includes White Irish, White European, Black, and Asian, with frequencies of 70 percent, 20 percent, 0 percent, and 10 percent respectively. Marital status shows 80 percent married, 20 percent cohabiting, and 0 percent single. Pregnancy status is split equally between nulliparous and parous at 50 percent each. Diagnoses include previous postpartum psychosis, schizophrenia, bipolar affective disorder, anorexia nervosa, perinatal obsessive-compulsive disorder, and previous birth trauma with secondary tokophobia, each with varying frequencies. PBPBM format is either in person or online, with 70 percent in person and 30 percent online. Partners attended in 60 percent of cases, and staff outside the perinatal mental health team attended in 30 percent of cases.

Navigate back to Table 1.

## Table 2 Long description

The table presents five themes and their corresponding subthemes. The first theme, Hoping for change, includes Experience motivating planning, Parenthood motivating planning, and Avoidance. The second theme, A wish to be heard, includes Valuing feeling listened to in the meeting, Written care plan carrying the woman’s voice, and Plan in the obstetric context. The third theme, Individualised care, includes Individualised care valued by women and Negatives of individualised care – burden and stigma. The fourth theme, Security of a plan in place, includes Making the plan, Specifics of the plan, and Timing of the meeting. The fifth theme, Role of the support network, includes Personal supports and Professional supports and communication.

Navigate back to Table 2.
