# Supplementary material for: ‘It won’t catch us off guard this time’: interview study exploring use of mental health care plans for birth and the postpartum period within perinatal mental health services
Source: BJPsych Open. 2026 Jun 24;12(4):e168. doi: 10.1192/bjo.2026.12019 (PMC13312273; doi:10.1192/bjo.2026.12019)
Supplement: Carey et al. supplementary material [file S2056472426120195sup001.docx]

**Supplementary material**

***‘It won't catch us off guard this time’:*** **An interview study exploring use of mental health care plans for birth and the postpartum period within perinatal mental health services.**

Cornelia Carey^1^, Rona Hunt^2^, Catherine Hinds^1^, Chaitra Jairaj^3^, Nuala Kane^4^

1. Perinatal Mental Health Service, National Maternity Hospital, Holles St, Dublin 02
2. Perinatal Mental Health Service, The Coombe Hospital, Cork St, Dublin 08
3. Department of Psychiatry, Trinity College Dublin, Dublin 02
4. Department of Psychiatry, University College Dublin, Belfield, Dublin 04

**Corresponding author:** Dr Nuala Kane, Department of Psychiatry, University College Dublin, Belfield, Dublin 04

**Supplementary material 1: Perinatal Mental Health Care Plan**

**Perinatal Mental Health Care Plan**

| Name |  |
| --- | --- |
| Date of Birth |  |
| Address |  |
| Telephone number |  |
| Estimated Date of Delivery |  |

| **Diagnosis** |  |
| --- | --- |
| **Medication** |  |
| **Perinatal Mental Health Service** |  |
| **Psychiatry Consultant** |  |
| **Obstetric Consultant** |  |
| **Perinatal MDT contact** |  |

| Relapse indicators |  |
| --- | --- |

| Antenatal Plan |  |
| --- | --- |
| Delivery Plan |  |
| Post-natal Plan |  |

| Crisis plan  for patient and family |  |
| --- | --- |

Useful contacts:

| Community Psychiatrist |  |
| --- | --- |
| Perinatal Mental Health |  |
| GP |  |
| NOK / family member |  |

**Supplementary material 2: Interview protocol for service users and supporters**

**Interview Protocol – service user interviews**

1. Introduction – explain interview process and purpose of interview. Explain what advance care planning is.
2. Could you tell me a bit about yourself and your experience of mental health difficulties? Your experience of mental health care?
3. Did you have any experience of advance care planning before the perinatal team suggested it?
4. What were your expectations of perinatal advance care planning? Did you feel it would be helpful? Did you have any concerns?
5. How did you find the pre-birth planning meeting?
   1. Were you happy with who was present?
   2. Did you feel that you could be open about your wishes and feelings? Did you feel listened to and included?
   3. Did you feel under pressure?
   4. Was there any part that you found distressing?
   5. What aspects of the meeting did you like? What didn't you like?
   6. How did you feel after the meeting?
6. What are your thoughts about the perinatal care plan?
   1. Which aspects of the care plan are most important to you?
   2. Were there any aspects of the care plan that you did not agree with (e.g., diagnosis, treatment, crisis plan)?
   3. Who do you feel that the care plan is for?
   4. How will you store it? Who has a copy?
7. Has making a care plan had any impact on you so far?
8. Do you think the care plan will make a difference for you when you give birth and afterwards? If so, how? If not, why?
9. Do you think the care plan will make a difference to you if you were to have a mental health crisis? If so, how? If not, why?
10. How might the perinatal care planning process be improved?

**Interview Protocol– family/ friend interviews**

1. Introduction – explain interview process and purpose of interview. Explain what advance care planning is.
2. Could you tell me a bit about yourself and your experience of being close to someone with mental health difficulties? Your experience of their mental health care?
3. Did you have any experience of advance care planning with your loved one before the perinatal team suggested it?
4. What were your expectations of perinatal advance care planning? Did you feel it would be helpful? Did you have any concerns?
5. How did you find the pre-birth planning meeting?
   1. Were you happy with who was present?
   2. Did you feel that you could be open about your wishes and feelings? Did you feel listened to and included?
   3. Did you feel under pressure?
   4. Was there any part that you found distressing?
   5. What aspects of the meeting did you like? What didn't you like?
   6. How did you feel after the meeting?
6. What are your thoughts about the perinatal care plan?
   1. Which aspects of the care plan are most important in your view?
   2. Were there any aspects of the care plan that you did not agree with (e.g., diagnosis, treatment, crisis plan)?
   3. Who do you feel that the care plan is for?
   4. Do you have a copy of the care plan?
7. Has making a care plan with your loved one had any impact on your loved on or on you so far?
8. Do you think the care plan will make a difference for your loved one when she gives birth and afterwards? If so, how? If not, why?
9. Do you think the care plan will make a difference to you and your partner if your partner were to have a mental health crisis? If so, how? If not, why?
10. How might the perinatal care planning process be improved?

**Supplementary material 3: Thematic Map with Subtheme Narrative and Quotes**

| **Themes** | **Subthemes** |
| --- | --- |
| 1. Hoping for change | Experience motivating planning |
|  | Parenthood motivating planning |
|  | Avoidance |
| 2. A wish to be heard | Valuing feeling listened to in the meeting |
|  | Written care plan carrying the woman”s voice |
|  | Plan in the obstetric context |
|  |  |
| 3. Individualised care | Individualised care valued by women |
|  | Negatives of individualised care – burden and stigma |
| The security of “a plan in place” | Making the plan |
|  | Specifics of the plan |
|  | Timing of the meeting |
|  |  |
| 5. Role of the support network. | Personal supports |
|  | Professional supports (and communication). |

| **Themes** | **Subthemes** | **Quotes** |
| --- | --- | --- |
| 1. Hoping for change | 1A. Experience motivating planning | - “It won’t catch us off guard this time”. (Partner 4, Postpartum psychosis) - “I just think I won’t be as afraid [this time], I thought I was going mad. I thought I was unfit to be a mother”. (SU 5, Perinatal OCD) - “That trauma has not got to do with this poor little baby. So, I was like, even just to make things better for that baby”(SU 6, Secondary tokophobia) - “You’re thinking back on your previous experience. OK well, this thing happened and maybe this time I’d like this instead. It’s causing you to think again, whereas you might not have done it in a long time, and it is a bit of a relief.” (SU 10, Secondary tokophobia) |
|  | 1B. Parenthood motivating planning | - “I was paranoid that he’d be taken from me…and that I wouldn’t be able to be with him. And I knew that in Ireland, there wasn’t... I know in England, when mothers become unwell, there [are] units where you can go into with your child.” (Participant 4, Postpartum psychosis) - “I have children [so] I would be reluctant for them to be around me if I was very unwell. I wouldn’t like them to be exposed to [that], as much as I love to see my kids and I found it very hard when my son was a baby and I was basically gone for seven weeks overnight and that was horrific. I would feel more guilt afterwards when I would be thinking “God you saw me unwell” and that would be harder to carry afterwards.” (Patient 2, Bipolar disorder) - “Just watching X going through it …wondering how long it would take to come back to herself. And then we had [the baby] as well.” (Partner 4, Postpartum psychosis) - “I just think I won’t be as afraid [this time], I thought I was going mad. I thought I was unfit to be a mother”. (SU 5, Perinatal OCD) |
|  | 1C. Avoidance | - “I was saying, I’m just trying not to think about it. And then she was saying, sometimes that isn’t a useful thing to do. So, I’ve been trying.” (SU 5, Perinatal OCD) - “And then you know you have to go back and discuss depression, medication, hypomania, which is crazy, right? So, you don't even want to talk about these things because you're not yourself. The care plan was very carefully discussed. I was told “Let's put it on paper and it's there. It doesn't mean that it's going to happen.” (SU 9, Bipolar disorder) - “And I just want to be, you know well and I want to be able to care for my baby” (SU 9, Bipolar disorder) |
| 2. A wish to be heard | 2a. Valuing feeling listened to in the meeting | - “Yes, they let us talk and let us speak and asked if we had any questions. It was a conversation-based meeting, not just listening to them.” (Partner 1, Schizophrenia) - “We both have our opinions, I think that they were both well received and listened to and there was real caregiving”. (Partner 7, Perinatal OCD) - “I actually found it nice and efficient and there is a point, and I also felt like she was offering information, but she was also listening to my perspective”. (Patient 7, Perinatal OCD) |
|  | 2b. Written care plan carrying the woman’s voice | - “It’s nearly like a will, what your wishes are, after the event”. (SU 2, Bipolar disorder) - “My husband was at the appointment, and he was given the information and the plan and it’s not that I want him to make a decision on my behalf, but he’s looking out for the signs.” (SU 7, Perinatal OCD) - “You can give it to your husband, you can give it to your midwife as well, and we will forward it to your doctor, to your midwife, to your GP. So, they all have it and it seems like I was really protected, right? So, if for example Doctor X is not available right? So, I can go to my GP, and she will have the same plan in front of her. Right? So, she knows what steps to take. If it’s Sunday or Saturday I have my husband there he can look at it and see what we have to do, and same applies if we have to go to emergency department. So, I feel like I have that support all around me which is great. So, everybody will have the same steps outlined there and I feel that I’m definitely not alone.” (SU 9, Bipolar disorder) - “You come into contact with so many different people when you’re in there, you’re in the A&E part, and then you’re up in the labour ward and there’s all different people and you feel you don’t have to keep explaining yourself.” (SU 10, Secondary tokophobia) - just the fact that you don’t have to advocate, you know, in that moment for yourself. (SU 10, Secondary tokophobia) |
|  | 2c. Plan in the obstetric context | - “It was very much what we just said to each other. It wasn’t anything different from her side, it was just what we had spoken about.” (SU 5, Perinatal OCD) - “They have the plans even post-delivery as well, so we believe in that.” (Partner 1, Schizophrenia) - “I was in with my consultant [obstetrician] today and she said we’ll make sure that this is done as well. I could even actually see in my notes today, like the care plan stuck in the front of it where it was said that it was going to be stuck.” (SU 6, Secondary tokophobia) - I feel that history was wiped now this file is just a brand-new blank file. But now I know that when they open the front page of my file a few lines down, it’s going to say the traumatic birth the first time. (SU 10, Secondary tokophobia) - “But the midwife and the doctor on the day, whether they will have time to read through the notes that we put there because there is a postnatal plan as well on medications, on the treatment, there are about 10 points there. So that would probably be my concern: whether it will be taken seriously by very busy staff on the day.” (SU 9, Bipolar disorder) |
| 3. Individualised care | 3a. Individualised care valued by women | - “I actually thought it was very good because it was very specific to me because I was afraid it would be very general” (SU 7, Perinatal OCD) - Wanting/not needing more certainty around specifics. Diagnostic clarity not important to everyone “But there’s overlap and they all kind of flow into one another but the fact that she’s being looked after is the important thing”. (Partner 7, Perinatal OCD) - “No, because you make it your own. You know, you tailor it yourself.” (SU 10, Secondary tokophobia) - “I’m quite happy with it, there’s nothing outrageous that I”m asking for.” (SU 10, Secondary tokophobia) |
|  | 3b. Negatives of individualised care – burden and stigma | - “They’ll think you’re odd you know if there’s that space in your chart that other people would see” (SU 5, Perinatal OCD) - “Obviously, there are people that are a lot worse than me and maybe still have an eating disorder very active at the moment, and I didn’t think that [it] would necessarily be the midwives” job to ensure that you’re eating and support you in that sense” (SU 8, Anorexia Nervosa) - “This sounds very strange, but I know that anyone I ask would be supportive and have no problem, but me as a person I think, I was really conscious of like I don’t want to bother someone. I don’t want to put their name on a list, and they’re worried thinking oh God if X gets sick what would we do, what would I do. Like that they have a sense of responsibility for me.” (SU 2, Postpartum psychosis) - “I find it’s hard to talk about some of those behaviours without feeling really embarrassed, but I didn’t feel embarrassed about it. She’s very easy to talk to.” (SU 5, Perinatal OCD) |
| 4. Security of ‘a plan in place’ | 4A. Making the plan | - “[It] kind of helps you relax, I think, just knowing that there’s a plan in place… After the meeting, like both myself and my husband came out with a weight off our shoulders.” (SU 4, Postpartum psychosis) - “… [I was] hoping for a plan for [the] middle ground of when things start to go rather than waiting until I need a full-blown medical admission.” (SU3, Anorexia Nervosa) - “I found even though we were talking about mental health planning it wasn’t frightening.**”** (SU 5, Perinatal OCD) - “She gave the heads up even before the meeting. We had a good idea about what was going to happen.” (partner 1, Schizophrenia) - “I wasn’t expecting it and found it a pleasant surprise.” (SU 8, Anorexia Nervosa) |
|  | 4B. Specifics of the plan | - “So, I just know that people will all be looking out for things and we included [early warning] signs to watch out for.” (SU 4, Postpartum psychosis) - “It’s like a security blanket for me,” (SU 4, Postpartum psychosis) - “[Medication is] nearly like an armour, you know that’s a protective thing stop me getting that unwell.” (SU 2, Bipolar disorder) - “I didn’t realise there’d be any kind of care planning, I was expecting the doctors to just guide me on the medication and what not and that was what I was concerned about.”(SU 8, Anorexia Nervosa) - “But ultimately what we’re doing right now is taking a wait and see approach which is to some people, not a big approach, but to me because I know that even in two weeks I wanted to ring up and try something, I have it all in place.”(SU 7, Perinatal OCD) |
|  | 4C. Timing of the meeting | - “I thought that was good that it was acknowledged and the meeting was brought forward a bit.” (SU 3, Anorexia Nervosa) - “I think it was requested that it would that be done a bit earlier just in light of it being an active eating disorder. So that would be my only addition in that it was kind of acknowledged and highlighted and it seems definitely agreed with it being sooner rather than later.” (SU 3, Anorexia Nervosa) - “I feel like maybe even just starting that a little bit earlier just to kind of have that like reassurance.” (SU 6, Secondary tokophobia) - “Ideally, I suppose maybe like round like 20 weeks. You know when you have your anatomy scan and you’re like, this is actually real now.” (Partner 7, Perinatal OCD) - “Say, after that meeting like I was really happy, so it was nice that they offered that and because I think if they didn’t offer that service, I maybe would have waited longer or would have gone into pregnancy being anxious, whereas I don’t feel anxious. (SU 4, Postpartum psychosis) - “Maybe even before the baby maybe if they plan something like this it would be more helpful. Like a long-term solution.” (Partner 1, Schizophrenia) - “I had a meeting with Doctor X when we were planning for the baby, So that was nice because we just had a lovely chat, but she outlined obviously risks such as postnatal depression, postnatal psychosis, so that I am aware that that can happen, that I have increased chances of having that because of my illness. But again, everybody is different, so it might happen. It might not happen, but it’s just good to know.” (SU 9, Bipolar disorder) |
| 5. Role of the support network | 5A. Personal supports | - “It was just myself. So, my husband is a bit reluctant to be involved. I feel for him because it would have been very difficult for him when I ended up in the hospital the last time.” (SU 2, Bipolar disorder) - “My partner actually wanted to come to it, and I was like there’s no need, you’ll be laughed at or told not to come in.” (SU 8, Anorexia Nervosa) - “At the time, obviously it was all about [my partner] but Dr X turned around to me and said how, how do you feel or how did you find it all you know which is kind of nice.” (partner 4, Postpartum psychosis) - “My husband knows where it is so he can always, you know check it and basically see if something is happening” & “He was there when I was unwell. He really supported me during that time, and he was actually very interested in having that plan so that he can refer to it.” (SU 9, Bipolar disorder) - “[And my partner was there as well and she’d ask him and it’s like it’s written that you think that would help, and there’s different things like that I wouldn’t have thought, and he could have said so that was really positive having him.](https://onedrive.live.com/?cid=6B1C458C5188A46F&id=6B1C458C5188A46F!sd9cd759d15194dcd81ad618bcd5f2af9)” (SU 7, Perinatal OCD) |
|  | 5B. Professional supports (and communication) | - “I thought it was better the midwife knows I’m having some problems. They told me they will help if any problems come up.” (SU 1, Schizophrenia) - “to have people in the same room and just make sure that everyone’s on the same page.” (SU 3, Anorexia Nervosa). - “They’re aware and I guess awareness is a great tool in itself (SU 3, Anorexia Nervosa) - “[I just feel like it is invisible enough sometimes in the, like, general clinics, I think there could be more first line stuff. Just a bit more awareness.”](https://onedrive.live.com/?cid=6B1C458C5188A46F&id=6B1C458C5188A46F!sbfb5242b941b49f1813398b68986a5e1) (SU 5, Perinatal OCD) |

| **Themes** | **Subthemes** |
| --- | --- |
| 1. Hoping for change | Experience motivating planning |
|  | Parenthood motivating planning |
|  | Avoidance |
| 2. A wish to be heard | Valuing feeling listened to in the meeting |
|  | Written care plan carrying the woman”s voice |
|  | Plan in the obstetric context |
|  |  |
| 3. Individualised care | Individualised care valued by women |
|  | Negatives of individualised care – burden and stigma |
| The security of “a plan in place” | Making the plan |
|  | Specifics of the plan |
|  | Timing of the meeting |
|  |  |
| 5. Role of the support network. | Personal supports |
|  | Professional supports (and communication). |

| **Themes** | **Subthemes** | **Quotes** |
| --- | --- | --- |
| 1. Hoping for change | 1A. Experience motivating planning | - “It won’t catch us off guard this time”. (Partner 4, Postpartum psychosis) - “I just think I won’t be as afraid [this time], I thought I was going mad. I thought I was unfit to be a mother”. (SU 5, Perinatal OCD) - “That trauma has not got to do with this poor little baby. So, I was like, even just to make things better for that baby”(SU 6, Secondary tokophobia) - “You’re thinking back on your previous experience. OK well, this thing happened and maybe this time I’d like this instead. It’s causing you to think again, whereas you might not have done it in a long time, and it is a bit of a relief.” (SU 10, Secondary tokophobia) |
|  | 1B. Parenthood motivating planning | - “I was paranoid that he’d be taken from me…and that I wouldn’t be able to be with him. And I knew that in Ireland, there wasn’t... I know in England, when mothers become unwell, there [are] units where you can go into with your child.” (Participant 4, Postpartum psychosis) - “I have children [so] I would be reluctant for them to be around me if I was very unwell. I wouldn’t like them to be exposed to [that], as much as I love to see my kids and I found it very hard when my son was a baby and I was basically gone for seven weeks overnight and that was horrific. I would feel more guilt afterwards when I would be thinking “God you saw me unwell” and that would be harder to carry afterwards.” (Patient 2, Bipolar disorder) - “Just watching X going through it …wondering how long it would take to come back to herself. And then we had [the baby] as well.” (Partner 4, Postpartum psychosis) - “I just think I won’t be as afraid [this time], I thought I was going mad. I thought I was unfit to be a mother”. (SU 5, Perinatal OCD) |
|  | 1C. Avoidance | - “I was saying, I’m just trying not to think about it. And then she was saying, sometimes that isn’t a useful thing to do. So, I’ve been trying.” (SU 5, Perinatal OCD) - “And then you know you have to go back and discuss depression, medication, hypomania, which is crazy, right? So, you don't even want to talk about these things because you're not yourself. The care plan was very carefully discussed. I was told “Let's put it on paper and it's there. It doesn't mean that it's going to happen.” (SU 9, Bipolar disorder) - “And I just want to be, you know well and I want to be able to care for my baby.” (SU 9, Bipolar disorder) |
| 2. A wish to be heard | 2a. Valuing feeling listened to in the meeting | - “Yes, they let us talk and let us speak and asked if we had any questions. It was a conversation-based meeting, not just listening to them.” (Partner 1, Schizophrenia) - “We both have our opinions, I think that they were both well received and listened to and there was real caregiving”. (Partner 7, Perinatal OCD) - “I actually found it nice and efficient and there is a point, and I also felt like she was offering information, but she was also listening to my perspective”. (Patient 7, Perinatal OCD) |
|  | 2b. Written care plan carrying the woman”s voice | - “It’s nearly like a will, what your wishes are, after the event”. (SU 2, Bipolar disorder) - “My husband was at the appointment, and he was given the information and the plan and it’s not that I want him to make a decision on my behalf, but he’s looking out for the signs.” (SU 7, Perinatal OCD) - “You can give it to your husband, you can give it to your midwife as well, and we will forward it to your doctor, to your midwife, to your GP. So, they all have it and it seems like I was really protected, right? So, if for example Doctor X is not available right? So, I can go to my GP, and she will have the same plan in front of her. Right? So, she knows what steps to take. If it’s Sunday or Saturday I have my husband there he can look at it and see what we have to do and same applies if we have to go to emergency department. So, I feel like I have that support all around me which is great. So, everybody will have the same steps outlined there and I feel that I’m definitely not alone.” (SU 9, Bipolar disorder) - “You come into contact with so many different people when you’re in there, you’re in the A&E part, and then you’re up in the labour ward and there’s all different people and you feel you don’t have to keep explaining yourself .” (SU 10, Secondary tokophobia) - “Just the fact that you don’t have to advocate, you know, in that moment for yourself.” (SU 10, Secondary tokophobia) |
|  | 2c. Care plan in the obstetric context | - “It was very much what we just said to each other. It wasn’t anything different from her side, it was just what we had spoken about.” (SU 5, Perinatal OCD) - “They have the plans even post-delivery as well, so we believe in that.” (Partner 1, Schizophrenia) - “I was in with my consultant [obstetrician] today and she said we’ll make sure that this is done as well. I could even actually see in my notes today, like the care plan stuck in the front of it where it was said that it was going to be stuck.” (SU 6, Secondary tokophobia) - “I feel that history was wiped now this file is just a brand-new blank file. But now I know that when they open the front page of my file a few lines down, it’s going to say the traumatic birth the first time.” (SU 10, Secondary tokophobia) |
| 3. Individualised care | 3a. Individualised care valued by women | - “I actually thought it was very good because it was very specific to me because I was afraid it would be very general” (SU 7, Perinatal OCD) - “Wanting/not needing more certainty around specifics. Diagnostic clarity not important to everyone “But there’s overlap and they all kind of flow into one another but the fact that she’s being looked after is the important thing”. (Partner 7, Perinatal OCD) - “No, because you make it your own. You know, you tailor it yourself.” (SU 10, Secondary tokophobia) - “I’m quite happy with it, there’s nothing outrageous that I”m asking for.” (SU 10, Secondary tokophobia) |
|  | 3b. Downsides of individualised care – burden and stigma /awareness of time and resources constraints and concern about implementation | - “They’ll think you’re odd you know if there’s that space in your chart that other people would see” (SU 5, Perinatal OCD) - “Obviously, there are people that are a lot worse than me and maybe still have an eating disorder very active at the moment, and I didn’t think that [it] would necessarily be the midwives” job to ensure that you’re eating and support you in that sense” (SU 8, Anorexia Nervosa) - “This sounds very strange, but I know that anyone I ask would be supportive and have no problem, but me as a person I think, I was really conscious of like I don’t want to bother someone. I don’t want to put their name on a list, and they’re worried thinking oh God if X gets sick what would we do, what would I do. Like that they have a sense of responsibility for me.” (SU 2, Postpartum psychosis) - “I find it’s hard to talk about some of those behaviours without feeling really embarrassed, but I didn’t feel embarrassed about it. She’s very easy to talk to.” (SU 5, Perinatal OCD) - “But the midwife and the doctor on the day, whether they will have time to read through the notes that we put there because there is a postnatal plan as well on medications, on the treatment, there are about 10 points there. So that would probably be my concern: whether it will be taken seriously by very busy staff on the day.” (SU 9, Bipolar disorder) |
| 4. Security of “a plan in place” | 4A. Making the plan | - “Kind of helps you relax, I think just knowing that there’s a plan in place.” (SU 4, Postpartum psychosis) - “[It] kind of helps you relax, I think, just knowing that there’s a plan in place… After the meeting, like both myself and my husband came out with a weight off our shoulders.” (SU 4, Postpartum psychosis) - “… [I was] hoping for a plan for [the] middle ground of when things start to go rather than waiting until I need a full-blown medical admission.” (SU3, Anorexia Nervosa) - “I found even though we were talking about mental health planning it wasn’t frightening.**”** (SU 5, Perinatal OCD) - “She gave the heads up even before the meeting. We had a good idea about what was going to happen.” (partner 1, Schizophrenia) - “I wasn’t expecting it and found it a pleasant surprise.” (SU 8, Anorexia Nervosa) |
|  | 4B. Specifics of the plan | - “So, I just know that people will all be looking out for things and we included [early warning] signs to watch out for.” (SU 4, Postpartum psychosis) - “It’s like a security blanket for me,” (SU 4, Postpartum psychosis) - “[Medication is] nearly like an armour, you know that’s a protective thing stop me getting that unwell.” (SU 2, Bipolar disorder) - “I didn’t realise there’d be any kind of care planning, I was expecting the doctors to just guide me on the medication and what not and that was what I was concerned about.”(SU 8, Anorexia Nervosa) - “But ultimately what we’re doing right now is taking a wait and see approach which is to some people, not a big approach, but to me because I know that even in two weeks I wanted to ring up and try something, I have it all in place.”(SU 7, Perinatal OCD) |
|  | 4C. Timing of the meeting | - “I thought that was good that it was acknowledged and the meeting was brought forward a bit.” (SU 3, Anorexia Nervosa) - “I think it was requested that it would that be done a bit earlier just in light of it being an active eating disorder. So that would be my only addition in that it was kind of acknowledged and highlighted and it seems definitely agreed with it being sooner rather than later.” (SU 3, Anorexia Nervosa) - “I feel like maybe even just starting that a little bit earlier just to kind of have that like reassurance.” (SU 6, Secondary tokophobia) - “Ideally, I suppose maybe like round like 20 weeks. You know when you have your anatomy scan and you’re like, this is actually real now.” (Partner 7, Perinatal OCD) - “Say, after that meeting like I was really happy, so it was nice that they offered that and because I think if they didn’t offer that service, I maybe would have waited longer or would have gone into pregnancy being anxious, whereas I don’t feel anxious. (SU 4, Postpartum psychosis) - “Maybe even before the baby maybe if they plan something like this it would be more helpful. Like a long-term solution.” (Partner 1, Schizophrenia) - “I had a meeting with Doctor X when we were planning for the baby, So that was nice because we just had a lovely chat, but she outlined obviously risks such as postnatal depression, postnatal psychosis, so that I am aware that that can happen, that I have increased chances of having that because of my illness. But again, everybody is different, so it might happen. It might not happen, but it’s just good to know.” (Patient 9, Bipolar disorder) |
| 5. Role of the support network | 5A. Personal supports | - “It was just myself. So, my husband is a bit reluctant to be involved. I feel for him because it would have been very difficult for him when I ended up in the hospital the last time.” (SU 2, Bipolar disorder) - “My partner actually wanted to come to it, and I was like there’s no need, you’ll be laughed at or told not to come in.” (SU 8, Anorexia Nervosa) - “At the time, obviously it was all about [my partner] but Dr X turned around to me and said how, how do you feel or how did you find it all you know which is kind of nice.” (partner 4, Postpartum psychosis) - “My husband knows where it is so he can always, you know check it and basically see if something is happening” & “He was there when I was unwell. He really supported me during that time, and he was actually very interested in having that plan so that he can refer to it.” (SU 9, Bipolar disorder) - “[And my partner was there as well and she’d ask him and it’s like it’s written that you think that would help, and there’s different things like that I wouldn’t have thought, and he could have said so that was really positive having him.](https://onedrive.live.com/?cid=6B1C458C5188A46F&id=6B1C458C5188A46F!sd9cd759d15194dcd81ad618bcd5f2af9)” (SU 7, Perinatal OCD) |
|  | 5B. Professional supports (and communication) | - “I thought it was better the midwife knows I’m having some problems. They told me they will help if any problems come up.” (SU 1, Schizophrenia) - “to have people in the same room and just make sure that everyone’s on the same page.” (SU 3, Anorexia Nervosa). - “They’re aware and I guess awareness is a great tool in itself (SU 3, Anorexia Nervosa) - “[I just feel like it is invisible enough sometimes in the, like, general clinics, I think there could be more first line stuff. Just a bit more awareness.”](https://onedrive.live.com/?cid=6B1C458C5188A46F&id=6B1C458C5188A46F!sbfb5242b941b49f1813398b68986a5e1) (SU 5, Perinatal OCD) |
